# Metal biogeochemistry in constructed wetlands based on fluviatile sand and zeolite- and clinopyroxene-dominated lava sand

**DOI:** 10.1038/s41598-017-03055-7

**Published:** 2017-06-07

**Authors:** Jen-How Huang, Sonja Paul, Silke Mayer, Eloise Moradpour, Ralf Hasselbach, Reto Gieré, Christine Alewell

**Affiliations:** 1 Environmental Geosciences, University of Basel, CH-4056 Basel, Switzerland; 2 Entsorgungsverband Saar (EVS), Mainzer Str. 261, 66121 Saarbrücken, Germany; 3 Department of Earth and Environmental Science, University of Pennsylvania, Philadelphia, PA 19104-6316 USA

## Abstract

For the first time, speciation of Fe, Mn, Zn, Ni, Cu and Pb was determined along the profiles of 8 constructed wetlands (CWs) consisting of fluviatile sand (Fluv), clinopyroxene-dominated lava sand (Cl-LS) and zeolite-dominated lava sand (Ze-LS), aiming at quantifying metal behaviour in CWs and the impact caused by different filter materials. With the exception of Mn, which underwent reductive dissolution, CWs were sinks for the studied metals. Metal accumulation rates differed in the following order: Ze-LS ≥ Cl-LS > Fluv CWs, reflecting the highest metal adsorption capacity and the lowest hydraulic conductivity of Ze-LS. Sequential extraction data indicated the highest metal mobility (readily mobilised and adsorbed fractions summing up to ~60%) in Fluv CWs, implying a higher risk of metal release into adjacent environments if Fluv from CWs will be improperly disposed after usage. Zinc and Ni were transported into the deeper CW layers to a larger extent than Cu and Pb, reflecting adsorption affinity to all filter materials in the order of Pb > Cu > Zn > Ni. Therefore, understanding metal speciation and mobility in such materials is crucial when they are considered for application as filters in CWs.

## Introduction

Constructed wetland (CW) is a green treatment technology, which has been applied for remediation of the waste water of different origins for several decades^1–4^. Numerous studies in the past have focused on the treatment efficiencies of biological and chemical oxygen demand, total suspended solids, and carbon, nitrogen and phosphorus contents in CWs (e.g. references in Vymazal^2^; Liu *et al*.^4^ and Zhang *et al*.^5^), whereas investigations concerning treatment of metals in waste waters using CWs are much less common, apparently because metals are usually not the target for treating waste water^6, 7^. Metals are considered as the main potentially toxic compounds present in the water-soluble fraction of CWs^8^. Unlike organic contaminants, metals do not undergo biological degradation and may either enter the food chain or spread into sediments where they remain until the physical or chemical conditions change^9^. Therefore, metals retained in the filter materials may later become a public health concern, if the filtration bed would be excavated and disposed.

Previous studies of metal behaviour in CWs focussed primarily on the removal efficiency by comparing the concentrations of metals in inlet and outlet water^10–18^. The metal removal efficiency varied widely, depending on the metal, the concentration of metals in inlet water^17, 18^, operating conditions of CWs (e.g., hydraulic retention time^17, 18^), and plants growing in CWs^19^. The removal rates of metal(loid)s in CWs were observed to decrease in the order Hg > Mn > Fe = Cd > Pb = Cr > Zn = Cu > Al > Ni > As^19, 20^. Using vertical flow CW microcosms, Yadav *et al*.^18^ demonstrated that removal rates of 10 mg L^−1^ Cr and Ni in inlet water increased from ~80–85% to ~90–100% if hydraulic retention time was extended from 6 to 48 hours. However, if Cr and Ni concentrations in the inlet water increased to 20 mg L^−1^, removal rates were lowered by ~10–20%. Moreover, an increase in the thickness of the filter bed from 65 to 95 cm enhanced the Cr and Ni removal efficiency by ~5–15%. Plants growing in CWs usually contribute little to the removal of metals ^7, 11, 17, 21^ but in rare cases, they may accumulate most of the total inflow metals in CWs (e.g. up to 98% in *Eichhornia crassipes* ^22^). The decisive role of filter materials in the metal removal in CWs was highlighted by May and Edwards^11^, who reported greater load rate and removal efficiency of metals in CWs compared to natural wetlands. Still, it is not clear how different filter materials may quantitatively influence the removal efficiency of metals and which mechanism is responsible for such differences. Moreover, the mobility and speciation of metals along vertical CW profiles were scarcely investigated so that it is difficult to estimate the potential risk of metal release into surrounding environments once the filter materials will be disposed after usage.

For the first time, detailed investigations on the behaviour of Fe, Mn, Zn, Ni, Cu and Pb were carried out in eight CWs located close to each other in SW-Germany, in which three filter materials of very different physical, chemical and hydraulic properties were utilised as the filter bed. Our research objectives were (1) to understand the influence of different filter materials on metal removal efficiencies; (2) to quantify the sink and source function of CW at different profile depths and towards different metals; (3) to identify the potential binding mechanisms of metals to different filter materials; (4) to estimate the mobility of metals along the CW profiles; (5) to reveal potential risks of metal releases from the filter materials, and (6) to examine whether or not the amounts of metals accumulated in the CWs reflected wetland operation time. The results obained in this study deliver fundamental knowledge as well as valuable experiences, which will serve as reference for the planning and designing of future CWs aimed at removing metals in waste water, especially in developing countries or the BRISC states, where metal production, mining sites and mine wastes are important.

## Material and Methods

### Site Description

**Figure 1:**
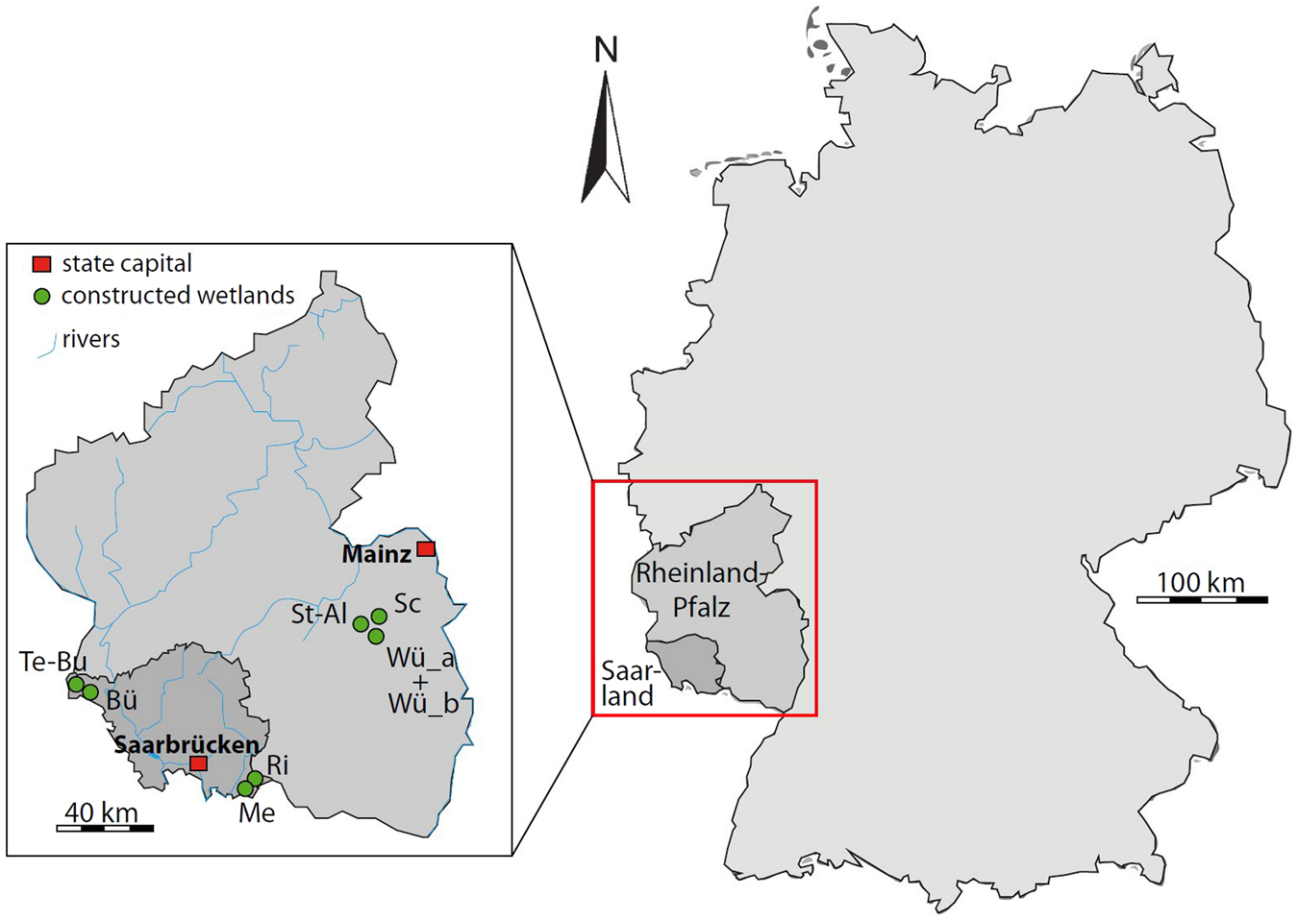
Location of constructed wetlands investigated in this study in southwestern Germany. Bü: Büschdorf; Me: Medelsheim; Ri: Riesweiler; Sc: Schneebergerhof; St-Al: St. Alban; Te-Bu: Tettingen-Butzdorf; Wü_a: Würzweiler 1; Wü_b: Würzweiler 2. This figure, inclusive of the map, was created by Silke Mayer using Adobe Illustrator cs6.

All investigated CWs are located in southwestern Germany, within 100 km from each other (Fig. 1), and are used as municipal plants, operating in vertical flow regime, to treat waste water. All CWs consist of a gravel layer at the bottom (25–30 cm thick, 4–8 mm grain size) and a filter layer (~70 cm thick) on the top. Three different filter materials were used: fluviatile sand (Fluv) in Schneebergerhof, St. Alban and Würzweiler, clinopyroxene-dominated lava sand (Cl-LS) in Medelsheim and Tettingen-Butzdorf, and zeolite-dominated lava sand (Ze-LS) in Büschdorf and Riesweiler. X-ray diffraction (XRD) analysis (AXS D8 XRD, Bruker, Karlsruhe, Germany, Fig. S1) showed that the Fluv were dominated by quartz, with minor amounts of feldspar (albite, microcline) and clay minerals (kaolinite, illite, chlorite and vermiculite, Fig. S1a). In addition to clinopyroxene, the Cl-LS also contained some olivine (forsterite), feldspathoid (leucite) and the zeolite mineral chabazite (Fig. S1b). The Ze-LS contained primarily the zeolites phillipsite and chabazite, but also some clinopyroxene, olivine (forsterite), feldspathoid (nepheline), and magnetite; in addition, quartz was identified, which suggests that this lava sand was mixed with quartz sand (Fig. S1c). Based on BET N_2_ analysis, the specific surface area of Fluv, Cl-LS and Ze-LS were ~1, 18 and 72–80 m^2^ g^−1^, respectively^23^. Waste water from small municipalities was fed onto the filter materials intermittently, with one week of loading followed by one week of resting, during which time the filters became dry and fully oxic again.

All facilities are connected to either a separated (in St. Alban and Medelsheim CWs) or a mixed sewer system (all other CWs), serving between 2.4–5 m^2^ per resident and having operating times between 5 and 15 years (Table S1). All CWs may have comparable loads of annual waste water input based on their almost identical municipal environment^24^. During CW operation, the substrate input remained similar, as evidenced by the litte variation of total organic carbon, phosphorus and NH_4_-N in the inflow of Riesweiler CW from 2007 to 2011^25^. All CWs were planted with reed (*Phragmites australis*) with a density of 4 plants per m^2^, but reed was partly displaced by stinging nettle (*Urtica dioica*). All facilities exhibit high removal rates for chemical oxygen demand, ammonia and phosphorous, which makes them suitable waste water facilities for local authorities^23, 26^. Operating conditions and more information concerning the investigated CWs are detailed in Table S1 and in Bruch *et al*.^27^, Hasselbach^24^ and Bruch *et al*.^23^. Hasselbach^24^ reported previously that the concentrations of Pb and Ni in the inlet and outlet waters of the studied CWs were below their ICP-OES detection limits (Pb < 20 µg L^−1^; Ni < 10 µg L^−1^). The concentrations of Cu averaged 16 µg L^−1^ in the inlet water of all CWs. However, Cu could be only occusionally found in the outlet water. Only Zn was typically detected, showing average concentrations of 110 and 20 µg L^−1^ in the inlet and outlet water, respectively thus yielding a removal efficiency of 81.1%^24^.

### Filter materials

At each CW, three profiles of filter material were sampled in June 2012 with a soil corer (diameter of 6 cm, length about 60 cm) with a distance of at least 1 m from each other. Each sample core was sliced into six vertical sections with a thickness of 10 cm. All samples were dried at 40 °C, then sieved to 5 mm, and milled for further analyses. Metal concentrations were also measured in the fresh starting materials of Ze-LS and Cl-LS, serving as the baseline values for estimating metal accumulation rates. Since certain Fluv CWs were in operation for 10 years at the time of this investigation (Table S1), no original starting materials were available for such analysis. Thus, we measured the metal concentrations in the deepest layer (at a depth of 50–60 cm) and regarded them as representative of the background values. The net accumulation rate of each metal in different CWs was calculated by dividing the concentration difference between CW layers and either the starting materials or the deepest layers by CW operating time. The densities of Fluv sand, Ze-LS and Cl-LS were all ~1.4 g cm^−3^.

### Sequential extraction

Sequential extraction in 2 g filter material was performed based on the schemes proposed by Tessier *et al*.^28^, which included the following four steps: (1) 8 mL of 1 M MgCl_2_, pH 7.0 at 20 °C for 1 h, representing the readily soluble fraction; (2) 8 mL of 1 M HOAc/NaOAc, pH 5.0 at 20 °C for 4 h, representing the strongly adsorbed fraction but less carbonate-associated, because of the exclusively low total inorganic C contents (all less than 0.8 wt%), which were detected in the filter materials during CW operation^26^; (3) 20 mL of 0.04 M NH_2_OH-HCl in 25% (v/v) HOAc at 96 °C, for 6 h, representing the reducible fraction; (4) 3 mL of 0.02 M HNO_3_ and 5 mL of 30% (w/w) H_2_O_2_, pH 2.0 (adjusted with HNO_3_), 85 °C for 2 h, then adding 3 mL of H_2_O_2_, pH 2.0, 85 °C for 3 h and finally mixing 20 mL of the dilute from 5 mL of 3.2 M NH_4_OAc in 20% (v/v) HNO_3_, for 0.5 h, representing the oxidisable fraction; (5) residue as the difference between the sum of the aforementioned four steps and the total metal concentrations determined according to the Swiss Soil Pollution Regulation^29^, in which the soil digestion for determination of total metal concentrations was recommend using 2 M HNO_3_ at 96 °C at a solid-to-solution ratio of 1:10 for 16 hours and with which 88–114% of metals from the certified materials LKSD-1 and LKSD-3 were recovered (Table S2).

### Batch adsorption experiments

Suspensions of Fluv (10 g L^−1^), Cl-LS and Ze-LS (5 g L^−1^ for both) in PIPES-buffered solutions (50 mM, pH 7.5) were allowed to equilibrate while stirring for 24 h. They were then spiked in a step-wise fashion with Zn, Ni, Cu and Pb to achieve final concentrations in the range 1–48 mg L^−1^ (details in Table 1). After each step, a 10 mL subsample was removed from the main vessel under vigorous stirring and pipetted into a 15 mL Falcon tube, which was placed on an over-head shaker for equilibration at room temperature for 24 h. Following equilibration, the solutions were syringe-filtered through 0.45 µm nitrocellulose filter membranes, and analysed for metal concentrations with ICP-OES (Vista-Pro radial, Varian, Germany). The adsorbed amounts of metals were calculated from the difference between the initial and final metal concentrations. Adsorption equilibria of Zn, Ni, Cu and Pb, obtained from the adsorption isotherms, were described by fitting a one-site Langmuir isotherm equation:

1 $${S}_{{\rm{eq}}}=\frac{{K}_{{\rm{L}}1}{b}_{{\rm{\max }}1}{C}_{{\rm{eq}}}}{1+{C}_{{\rm{eq}}}}$$

where *S*_eq_ (mg g^−1^) and *C*_eq_ (mg L^−1^) are the sorbed and corresponding dissolved concentrations, respectively, of Zn, Ni, Cu or Pb at equilibrium, *K*_L1_ (L mg^−1^) is the respective Langmuir sorption coefficient, and *b*_max1_ (mg g^−1^) is the maximum adsorption capacity for Zn, Ni, Cu or Pb.

**Table 1:** Parameters of one-site Langmuir adsorption isotherms (Eq. 1) fitted to experimental data for Zn, Ni, Cu and Pb adsorption to zeolite- (Ze-LS) and clinopyroxene-dominated lava sand (Cl-LS) and fluviatile sand (Fluv) (Fig. 4).

Zn Adsorption Isotherms				
Sand type/Concentrations	[Zn_total_] (mg L^−1^)	*K*_L1_ (L mg^–1^)	*b*_max1_ (mg g^–1^)	R^2^
Fluv/10 g L^−1^	2–13	0.51	0.19	0.97
Cl-LS/5 g L^−1^	2–13	0.45	1.19	0.95
Ze-LS/5 g L^−1^	7–17	0.55	2.22	0.94
**Ni Adsorption Isotherms**				
**Sand type/Concentrations**	**[Ni**_**total**_ **] (mg L** ^**−1**^ **)**	***K***_**L1**_ **(L mg** ^**–1**^ **)**	***b***_**max1**_ **(mg g** ^**–1**^ **)**	**R** ^**2**^
Fluv/10 g L^−1^	0.5–10	0.64	0.06	0.94
Cl-LS/5 g L^−1^	1–11	0.43	0.45	0.94
Ze-LS/5 g L^−1^	2–11	0.56	0.80	0.94
**Cu Adsorption Isotherms**				
**Sand type/Concentrations**	**[Cu**_**total**_ **] (mg L** ^**−1**^ **)**	***K***_**L1**_ **(L mg** ^**–1**^ **)**	***b***_**max1**_ **(mg g** ^**–1**^ **)**	**R** ^**2**^
Fluv/10 g L^−1^	2–13	1.33	3.10	0.96
Cl-LS/5 g L^−1^	2–13	2.23	35.6	0.82
Ze-LS/5 g L^−1^	7–25	3.17	570	0.92
**Pb Adsorption Isotherms**				
**Sand type/Concentrations**	**[Pb**_**total**_ **] (mg L** ^**−1**^ **)**	***K***_**L1**_ **(L mg** ^**–1**^ **)**	***b***_**max1**_ **(mg g** ^**–1**^ **)**	**R** ^**2**^
Fluv/10 g L^−1^	2–12	0.19	1.75	0.68
Cl-LS/ 5 g L^−1^	10–25	0.35	10.8	0.86
Ze-LS/5 g L^−1^	15–48	—	—	—

—: Not available.

## Results

### Metal concentrations along the profiles of different constructed wetlands

**Figure 2:**
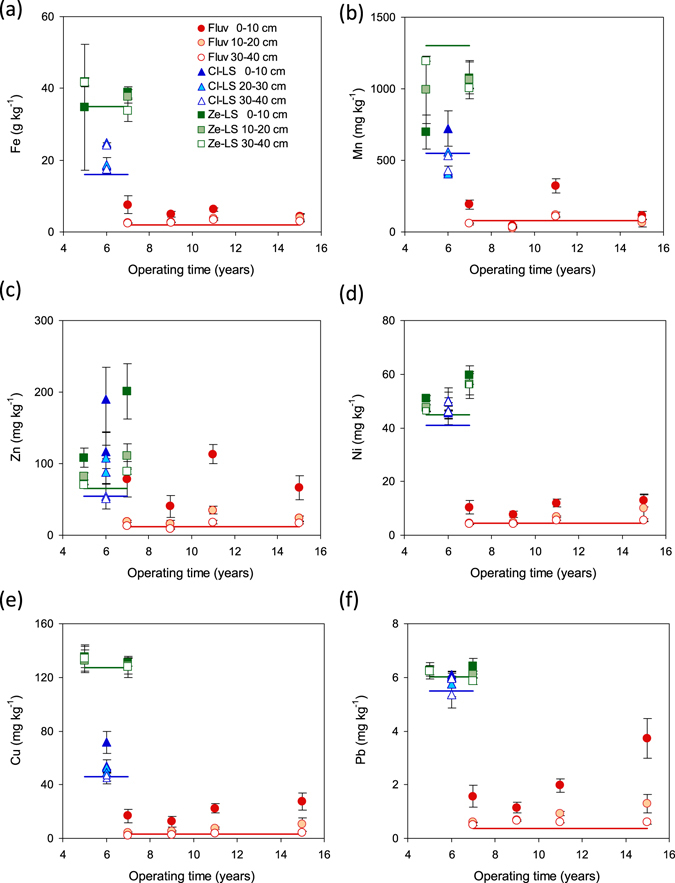
Concentrations of (**a**) Fe, (**b**) Mn, (**c**) Zn, (**d**) Ni, (**e**) Cu and (**f**) Pb at different depths of fluviatile sand (Fluv) and clinopyroxene- (Cl-LS) and zeolite-dominated lava sand (Ze-LS)-based constructed wetland profiles of different operation time. Solid lines show the baseline concentrations of metals in the starting materials of both lava sands and in the deepest layers of fluviatile sand based constructed wetlands.

In general, the concentrations of all metals analysed during this investigation are decreasing with depth in the studied Fluv CWs (Fig. 2). The same tendencies are also observed for the Cl-LS and the Ze-LS CWs, except for Mn, which does not exhibit such a clear trend. Individual metal abundances were in the order Fe > Mn > Zn ≥ Cu ≥ Ni > Pb, and the total metal contents were in the order Ze-LS ≥ Cl-LS > Fluv CWs, which reflects the background concentrations in the filter materials (Fig. 2). The concentrations of Fe were highest in Ze-LS CWs, with values of ~40 g kg^−1^, followed by Cl-LS CWs (18–25 g kg^−1^) (Fig. 2a). The lowest concentrations of Fe were found in Fluv CWs (2.4–7.6 g kg^−1^), reflecting the predominance of quartz in Fluv (Fig. S1a). Calculations based on the difference in concentration of Fe in CW and original filter materials revealed an annual Fe net accumulation of 60.6–137 g m^−2^ yr^−1^ in Fluv CWs, 209–795 g m^−2^ yr^−1^ in Cl-LS CWs, and 123–560 g m^−2^ yr^−1^ in Ze-LS CWs (Table S3–1). The concentrations of Mn in our CWs were highest in Ze-LS (697–1200 mg kg^−1^), decreasing to Cl-LS (408–723 mg kg^−1^) and Fluv CWs (26–192 mg kg^−1^) (Fig. 2b). Higher Mn concentrations in the surface compared to the deeper layers were only observed in two Fluv CW profiles (Würzweiler 1 and 2). Conversely, there were higher Mn concentrations in the deeper layers than in the surface layers in Ze-LS CWs. It is of note that three of the four studied Fluv CWs showed net Mn accumulation (219–4400 mg m^−2^ yr^−1^), whereas most lava sand-based CWs showed net release of Mn (−12100 to −20500 mg m^−2^ yr^−1^) (Table S3–2). The concentrations of Zn in Cl-LS and Ze-LS CWs ranged between 51 and 200 mg kg^−1^ and were mostly higher than those in Fluv CWs (up to ~113 mg kg^−1^) (Fig. 2c). Among Zn, Ni, Cu and Pb, Zn had the most elevated concentrations in CW compared to the original filter materials, resulting in highest net accumulation rates of 186–1560, 2560–5060 and 2170–4820 mg m^−2^ yr^−1^ in Fluv, Cl-LS and Ze-LS CWs, respectively (Table S3–3). Nickel concentrations in Cl-LS and Ze-LS CWs were higher (46–60 mg kg^−1^) than those in Fluv CWs (4–13 mg kg^−1^) (Fig. 2d), with net accumulation rates of 473–755 and 316–961 mg m^−2^ yr^−1^ in Cl-LS and Ze-LS CWs, respectively, and 18.4–154 mg m^−2^ yr^−1^ in Fluv CWs (Table S3–4). The concentrations of Cu were highest in Ze-LS (121–135 mg kg^−1^) and decreased to Cl-LS (45–72 mg kg^−1^) and Fluv CWs (1.9–28 mg kg^−1^) (Fig. 2e). The corresponding accumulation rates were 187–348 mg m^−2^ yr^−1^ in Fluv CWs, 338–787 mg m^−2^ yr^−1^ in Cl-LS, and 159–751 mg m^−2^ y^−1^ in Ze-LS CWs (Table S3–5). Lead showed higher concentrations in Cl-LS and Ze-LS CWs (5.36–6.42 mg kg^−1^) than in Fluv CWs (0.5–4 mg kg^−1^), but its concentrations were 1–2 orders of magnitude lower than those of Ni, Cu and Zn (Fig. 2f). Moreover, Pb showed much lower net accumulation rates in all CWs (25.6–136 mg m^−2^ yr^−1^, Table S3–6).

Concentrations of Zn and Ni at 10–30 cm depth as compared to the baseline concentrations, i.e. those in the original filter material (or the concentrations in the deepest layer in the case of Fluv CWs), were considerably more enhanced than those of Pb and Cu in all CWs (Fig. 2). For the Fluv CWs, the Zn concentrations at 10–20 cm depth seem to increase with operating time, but the correlation is poor (Figs 2c and S2c). Very good positive correlations between concentration in Fluv CWs and time of operation, however, are found for Ni (bottom two layers), Cu (all depths), and Pb (top two layers; see Figs 2d,e,f and S2d,e,f), allowing for estimation of annual net accumulation. Linear regression revealed Cu accumulation rates of 1.65 and 0.80 mg kg^−1^ yr^−1^ in the surface layer and at 10–20 cm depth, respectively (Fig. S2e), which were comparable with those calculated on the basis of concentration difference (1.06–1.97 and 0.16–0.49 mg kg^−1^ yr^−1^, respectively; Table S4–5). In the case of Pb, linear regression revealed 0.30 and 0.09 mg kg^−1^ yr^−1^ of Pb accumulation in the surface layer and at 10–20 cm depth, whereas the concentration difference method yielded 0.08–0.22 and 0.03–0.06 mg kg^−1^ yr^−1^ (Table S4–6).

### Sequential extraction of heavy metals in the filter materials of constructed wetlands

**Figure 3:**
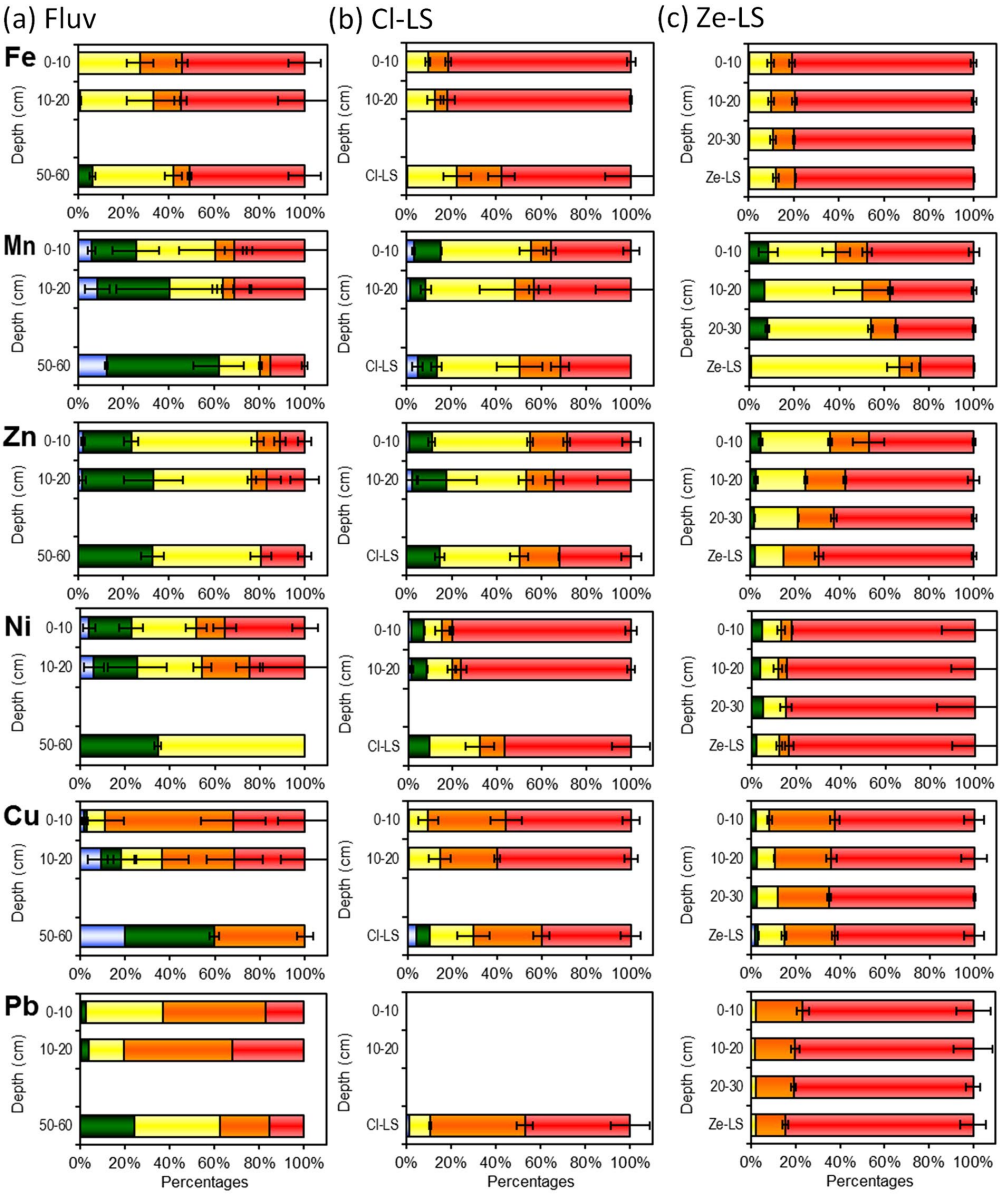
Relative distribution of sequential extraction-based fractions of Fe, Mn, Zn, Ni, Cu and Pb at different depths and in the corresponding starting materials of constructed wetland profiles based on (**a**) fluviatile sand (Fluv) (**b**) clinopyroxene-dominated lava sand (Cl-LS) and (**c**) zeolite-dominated lava sand (Ze-LS). : readily soluble fraction; : strongly adsorbed fraction; : reducible fraction; : oxdisible fraction; : residual fraction. Mean values and standard deviations of 2 replicates of each site together with 4 site replicates for fluviatile sites and 2 site replicates for lava sand sites are shown.

The metal distribution in different sequential extraction-based fractions of the CW filter materials were dependent on the metal, the filter material and the depth within the CW profiles (Fig. 3). The association of Fe, Zn, Ni, Cu and Pb with the residual fraction was markedly different in the three types of sand, ranging from Ze-LS CW (50–85%) to Cl-LS (30–80%) and to Fluv CW (11–55%). Since the aforementioned differences can be clearly observed already in the deepest Fluv layers and in the original filter materials, this result can be attributed to the different mineralogical composition of the original materials (see XRD analysis, Fig. S1). Generally, the proportions of metals found in the readily soluble, and thus mobile, fractions were in the order Fluv > Cl-LS > Ze-LS CWs. The highest proportions of readily soluble fractions were observed for Mn, Ni and Cu in Fluv CWs (3.9–20%). In comparison, only very small or negligible proportions of these metals can be readily mobilised in Cl-LS CWs (1.3–3.6%) or in Ze-LS CWs, respectively (Fig. 3b,c). For example in the deepest layer of the Fluv CWs, 12% of Mn and 20% of Cu was readily mobilised (Fig. 3a), whereas in the original filter materials of the Cl-LS only 5.1% of Mn and 3.8% of Cu were mobilised (Fig. 3b).

The Fe distribution among different fractions in Ze-LS remained nearly the same along the profile, whereas the reducible fraction of Fe increased with depth in the Cl-LS and Fluv materials (Fig. 3). The reducible fraction of Mn increases considerably with depth in Ze-LS and remains roughly unchanged in Cl-LS CWs. On the other hand, it decreased in Fluv, whereas the strongly adsorbed fraction increased considerably with depth (Fig. 3a). In Fluv and Cl-LS CWs, the majority of Zn was present in the reducible fraction (~40–60% of total Zn) (Fig. 3a,b), whereas residual Zn was the dominant form in the Ze-LS (30–40%). In Fluv CWs, strongly adsorbed Zn accounted for 10–40% of total Zn and was the second most important fraction. Noticeably, the proportion of reducible Zn in the Ze-LS CW profiles decreased with the depth from 35% to 20% (Fig. 3c). The majority of Ni was in the residual fraction of both the Cl-LS and Ze-LS (70–85%, Fig. 3b,c), in contrast to the distribution in Fluv. Independent of the type of filter materials, a large proportion of both Cu and Pb is present in oxidisable form (Fig. 3), especially in the surface layers. These oxidisable fractions account for 25–30% of total Cu and 20% of total Pb in Ze-LS and Cl-LS CWs and 30–50% of total Cu and 50% of total Pb in Fluv CWs. These oxidisable fractions of Cu and Pb in Cl-LS and Ze-LS CWs were all significantly higher than those in the original filter materials, especially in the surface layer.

### Adsorption behaviour of heavy metals to different filter materials

**Figure 4:**
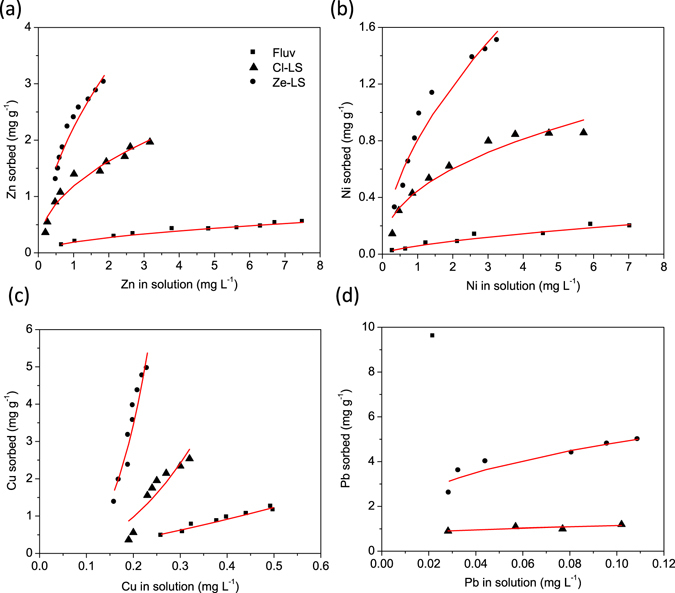
Adsorption isotherms of (**a**) Zn, (**b**) Ni, (**c**) Cu and (**d**) Pb to fluviatile sand (Fluv) and zeolite- (Ze-LS) and clinopyroxene-dominated lava sand (Cl-LS). Red lines show the fitting results using a one-site Langmuir adsorption isotherm.

Adsorption isotherms indicated that the adsorption affinity of Pb, Cu, Zn and Ni to different filter materials generally is in the order Ze-LS > Cl-LS > Fluv (Fig. 4). Based on the adsorption isotherms, Pb adsorbed most strongly to all filter materials, followed by Cu (Fig. 4c,d). Adsorption of Zn and Ni was markedly weaker than that of Cu and Pb (Fig. 4a,b). The maximum adsorption capacity, obtained from fitting with the Langmuir equation, was observed for Cu, which had a value of 570 mg g^−1^ in Ze-LS (Table 1). The maximum adsorption capacity of Cu was one order of magnitude lower in Cl-LS (35.6 mg g^−1^), and only 3.10 mg g^−1^ in Fluv. In comparison, the maximum adsorption capacities of Zn and Ni were ≤2.22 and ≤0.80 mg g^−1^, respectively (Table 1), with the same tendency of Ze-LS > Cl-LS > Fluv.

Fitting of the Pb adsorption isotherms with the Langmuir equation was less successful (Fig. 4d and Table 1). Namely, the very strong Pb adsorption to all filter materials did not allow us to assess an adsorption isotherm as precisely as for the other metals. Even when Pb concentrations as high as 48 mg L^−1^ were applied during the adsorption experiments, Pb concentrations in solution were still close to or below our instrumental detection limits (0.01 mg L^−1^). Since the mineralogical composition and grain size of individual particles of the three filter materials were all highly heterogeneous, it is not feasible to perform the adsorption experiments with smaller amounts of sorbents. Higher Pb concentrations were not considered to avoid either Pb precipitation or over-acidification of the batch solution.

### Estimation of metal budget in constructed wetlands

With the exception of Mn, which shows net releases from the filters, the total theoretical adsorption capacity estimated using the Langmuir equation of each CW for Zn, Ni and Cu was much larger than the annual accumulation rate of these metals (Tables 1 and S3). Taking the annual accumulation rates as estimates, saturation of the total adsorption capacity would require 50–50000 years, depending on the filter materials and metals. Zinc was predicted to reach its adsorption saturation most rapidly in Fluv CWs (57.6–234 years). In comparison, reaching adsorption saturation of Zn is expected to take 132–260 and 258–574 years in Ze-LS and Cl-LS CWs, respectively. The slowest to reach saturation was the adsorption of Cu to Ze-LS (25300–58900 years). The years required to reach adsorption saturation among different filter materials is generally in the order of Ze-LS > Cl-LS > Fluv CWs.

## Discussion

Elevated concentrations in most filter materials in the investigated CWs as compared to the original materials or the deepest Fluv layers indicated that all CWs are, in general, sinks for Fe, Zn, Ni, Cu and Pb. The annual accumulation rates of Zn, Ni, Cu and Pb, in the general order of Ze-LS ≥ Cl-LS > Fluv CWs, were in good agreement with their adsorption behaviour to the different filter materials: While the adsorption isotherms indicated highest adsorption affinity and capacity of metals to Ze-LS (Fig. 4 and Table 1), sequential extraction revealed that the largest proportion of readily soluble metals is found in Fluv CWs (Fig. 3). The much higher specific surface area of Ze-LS (~72–80 m^2^ g^−1^) compared to that of Cl-LS (~18 m^2^ g^−1^) and Fluv (~1 m^2^ g^−1^) explains only partly the tremendously high removal efficiency of metals in Ze-LS CWs. The high removal efficiency of metals is also attributed to the low hydraulic conductivity due to the shrinking and swelling capacity of the zeolite^23, 27^. Zeolites not only increased the metal sorption capacity but they are also able to adsorb large amounts of water, prolonging the equilibration time of metals at the filter solid-water interface^23, 27^.

The metal levels and their annual accumulation rates in CWs in the order of Zn ≫ Ni > Cu > Pb more likely reflected their environmental abundance instead of the adsorption affinity. Although Zn has a lower adsorption affinity to filter materials than Cu and Pb, its concentrations and accumulation rates were highest in all CWs (excluding Fe and Mn), reflecting its environmental abundance^30, 31^. Subsequently, the very low input of Pb into CWs^6, 24^ explained its comparatively low concentrations and accumulation rates in all CWs even with the highest adsorption affinity. Different adsorption affinities among Zn, Ni, Cu and Pb to the filter materials were best mirrored in the vertical distribution of these metals in the CW profiles, in which considerable amounts of Zn and Ni, but only trace amounts of Cu and Pb, were accumulated at a depth of 10–30 cm depth (Fig. 2). In addition, the extent of the decrease in concentrations and annual accumulation rates with depth were in the order Pb ≥ Cu > Zn ≥ Ni (Table S4). These results altogether allow us conclude that the vertical transport of the studied metals in the CWs has the general order Ni ≥ Zn > Cu ≥ Pb, which is consistent with the order of metal mobility and bioavailability proposed by Lee *et al*.^32^, Irvine *et al*.^33^ and Sheoran *et al*.^34^. Utilising linear regression to estimate accumulation rates in Fluv CWs succeeded only for Cu and Pb but failed for Zn and Ni. This finding may also support the stronger adsorption of Cu and Pb than Zn and Ni to Fluv so that the retention of Cu and Pb in the CWs was comparatively less influenced by the minor heterogeneity among different Fluv CWs (Table S1). Metal concentrations in sediments did not reflect the time of operation among 7 crushed rock- or gravel-based CWs in Czech Republic^6^. Increased mass of the filter bed caused by the sedimentation of suspended solids in waste water was indicated as the major reason for the aforementied observation. Thus, the significant correlations between Cu and Pb concentrations and the operating time among our Fluv CWs indicated the effectiveness of sedimentation ponds at our sites to eliminate suspended particles in waste water^27^.

Remarkable losses of Mn in Ze-LS and Cl-LS CWs and the large pools of readily mobilisable Mn revealed the potential of CWs as a source of Mn. Loss of Mn during CW operation appears to be associated with anoxia or, more concretely, reductive dissolution of Mn oxides^35, 36^. Losses of Mn from Fluv CWs was less pronounced, probably due to the lower background Mn concentration (~80 mg kg^−1^) and the low abundance of reducible Mn in Fluv (14%) as compared to Cl-LS and Ze-LS (~550 and ~1300 mg kg^−1^ and 37 and 66%) (Figs 2b and 3c). Thus, the inlet input of Mn may have a larger effect than reductive dissolution. Like Mn oxides, iron (hydr)oxides may undergo reductive dissolution under anoxic conditions^35, 36^. However, we observed accumulation of Fe in all types of filter materials. Reduction of Fe (hydr)oxides is usually less favoured compared to that of Mn oxides due to the lower redox potential of Fe (hydr)oxides than Mn oxides^35, 37^. In addition, there may be effective formation of secondary Fe(II) minerals^38–40^, thus trapping Fe(II) released into the solution. The aforementioned hypothesis especially relates to the Ze-LS CWs with largest Mn net release (Tables S3 and S4). Namely the proportion of reducible Mn in Ze-LS decreased during CW operation, whereas the proportion of reducible Fe did not change significantly (revealed by comparing their proportions in CW and original filter materials or deepest Fluv layers, Fig. 3c).

**Figure 5:**
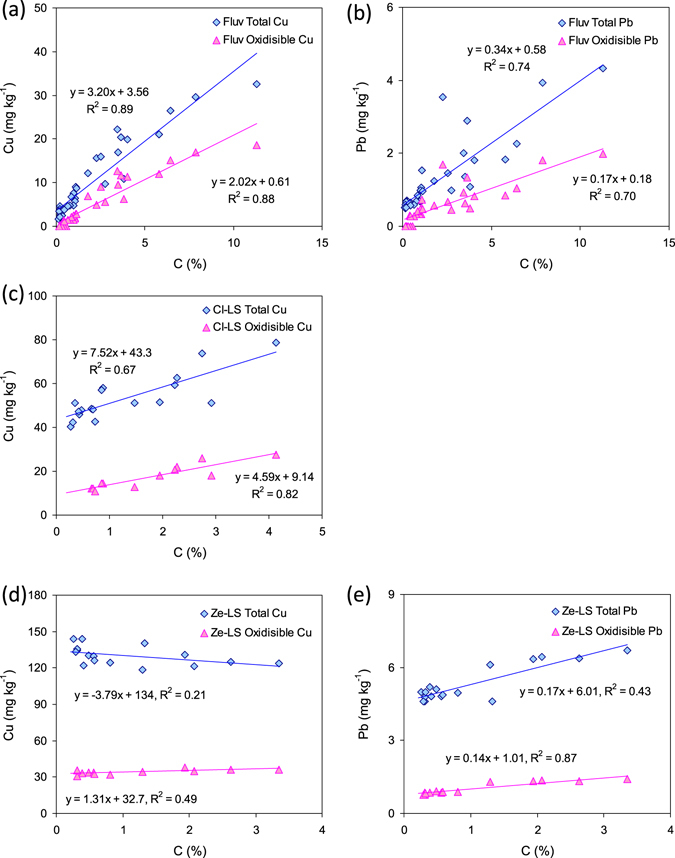
Correlations between carbon contents and total and oxidisible Cu and Pb in the filter materials of constructed wetlands based on (**a**,**b**) fluviatile sand (Fluv), (**c**) clinopyroxene- (Cl-LS) and (**d**,**e**) zeolite-dominated lava sand (Ze-LS).

The variation of metal proportions in different sequential extraction-based fractions in filter materials during CW operation may deliver information about retention or release mechanisms of metals in CWs. Decreased proportions of reducible Mn indicated reductive dissolution during CW operation, whereas increased association of Zn with reducible phases may imply Zn adsorption to Mn and Fe (hydr)oxides as the major mechanism of removal, especially in the surface layer. Manganese and Fe (hydr)oxides are effective sorbents for Zn^41, 42^. Such a process is most likely to occur in Ze-LS CWs, as Ze-LS contained the highest level of Fe among three filter materials. Another noticeable change of metal distribution during CW operation is seen for Pb and Cu, showing increased association with oxidisable phases, which in many cases are organic matter and sulphides^28^. The strong association of Pb and Cu with organic matter in soils and sediments is well-known^43, 44^ and is especially plausible in our Cl-LS and Ze-LS CWs, as indicated by the markedly improved correlations with C when replacing total Pb (insignificant for Ze-LS) and Cu (r = 0.82, p = 0.002 for Cl-LS; insignificant for Ze-LS) with oxdisible Pb (r = 93, p < 0.001 for Ze-LS) and Cu (r = 0.91, p < 0.001 and r = 0.70, p = 0.04 for Cl-LS and Ze-LS, respectively) (Fig. 5). Such an improvement was not observed in Fluv CWs, probably as a result of the predominance of oxidisable Pb and Cu (Fig. 3a). Although precipitation of Pb and Cu sulphides is possible during operation of CWs (redox potential may reach <−500 mV and pH ranged 7.5–8.5^24, 27^, long-term accumulation of Pb and Cu associated with sulphides seemed little due to weekly exchanged anoxic and oxic cycles in all CWs.

Plants are capable of taking up metals and changing metal speciation in soils^7, 11, 17, 21^. However, the influence of such plant-metal interaction on metal biogeochemistry in our CWs is almost negligible due to the very low mass of the plants. Taking the metal concentrations in reed and stinging nettle from our preliminary study as representative^26^, the annual loss of all studied metals caused by plant uptake was less than 1% of the annual metal input. Reed is known to pump oxygen into the rhizosphere via aerenchyma^45^, and thus influences redox conditions and metal speciation in soils. Nonetheless, such an effect appears to be insignificant in our CWs, because we still observed considerable decreases in Mn concentration caused by reductive dissolution (Table S3–2).

According to current metal inputs into the studied CWs, saturation of their retention capacity will take at least 50, 130 and 250 years in Fluv, Ze-LS and Cl-LS CWs, respectively. Our study shows clearly that the Ze-LS CWs outperform the Cl-LS and Fluv CWs in terms of accumulating and retaining metals due to their high adsorption capacity and lower hydraulic conductivity. On the contrary, in Fluv CWs, the risk of metal release was higher due to their larger pool of readily mobilisable metals. Our study on metal budgets and sequential extraction-based fractionation at different CW depths also highlighted that the metal removal efficiency of a CW is not only determined by its retention capacity but also strongly influenced by both the metal concentration and speciation in the filter material. Thus, these two parameters should be also assessed when considering a filter material for a CW.

## Electronic supplementary material

Supplementary Information
