# Supplementary material for: Metal biogeochemistry in constructed wetlands based on fluviatile sand and zeolite- and clinopyroxene-dominated lava sand
Source: Sci Rep. 2017 Jun 7;7:2981. doi: 10.1038/s41598-017-03055-7 (PMC5462811; doi:10.1038/s41598-017-03055-7)
Supplement: Supplementary file 1 — Supplementary Information [file 41598_2017_3055_MOESM1_ESM.doc]

**Supplementary Materials**

***Scientific Reports***

Metal biogeochemistry in constructed wetlands based on fluviatile sand and zeolite- and clinopyroxene-dominated lava sand

Jen-How Huang^1^, Sonja Paul^1^, Silke Mayer^1^, Eloise Moradpour^1^, Ralf Hasselbach^2^, Reto Gieré^3^, Christine Alewell^1^

*1. Environmental Geosciences, University of Basel, CH-4056, Basel, Switzerland*

*2. Entsorgungsverband Saar (EVS), Mainzer Str. 261, 66121 Saarbrücken, Germany*

*3. Department of Earth and Environmental Science, University of Pennsylvania, Philadelphia, PA 19104-6316, USA*

*Corresponding author. Tel.: +41 61 2070483; fax: +41 61 2670479.

E-mail address:[jen-how.huang@unibas.ch](mailto:jen-how.huang@unibas.ch)

**3 Tables, 3 Figures, 10 pages**


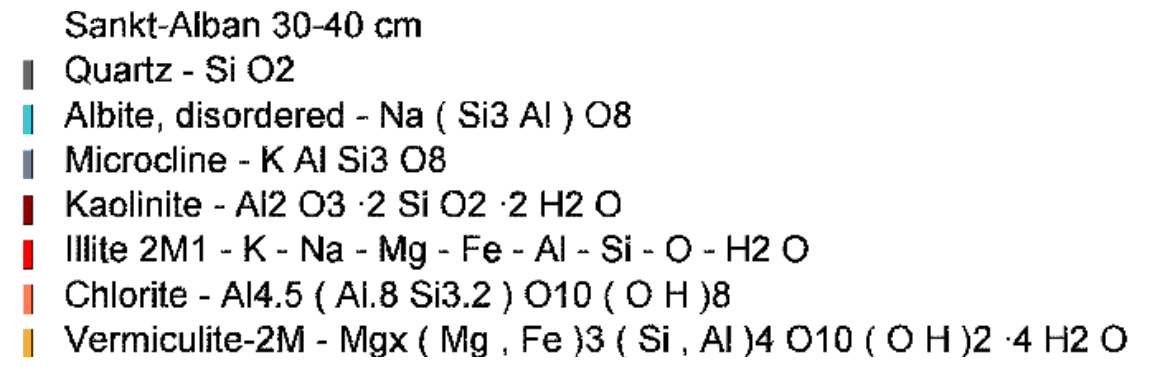


**Fig. S1a.** XRD diagram for fluviatile sand used as filter material in St. Alban constructed wetland. Quartz is the predominant mineral. Minor minerals present are mostly feldspars (albite, microcline), with trace amounts of clay minerals (kaolinite, illite, chlorite and vermiculite).

**Fig. S1b.** XRD diagram for clinopyroxene-dominated lava sand used as filter material in Meddelsheim constructed wetland. Clinopyroxene is clearly the dominant mineral phase. Minor olivine (forsterite), leucite (feldspathoid), and chabazite (zeolite) were also identified.

**Fig. S1c**. XRD diagram for zeolite-dominated lava sand used as filter material in Büschdorf constructed wetland. Phillipsite and chabazite were identified as zeolite minerals. Nepheline, clinopyroxene, olivine (forsterite), and magentite were are also identified. The presence of quartz indicates that the lava sand was mixed with quartz sand.

**Table S1.** Characteristics and operation conditions of the investigated constructed wetlands.

|  | Schnee-bergerhof | St.Alban | Würzweiler 1 | Würzweiler 2 | Medelsheim | Tettingen-Butzdorf | Büschdorf | Riesweiler |
| --- | --- | --- | --- | --- | --- | --- | --- | --- |
| **Basic Information** |  |  |  |  |  |  |  |  |
| Start of operation | 1997 | 2003 | 2001 | 2005 | 2006 | 2006 | 2005 | 2007 |
| Length of operation (yr) | 15 | 9 | 11 | 7 | 6 | 6 | 7 | 5 |
| Designed PE^a^ | 120 | 1150 | 250 | 300 | 500 | 500 | 310 | 100 |
| Area (m^2^) | 600 | 2760 | 625 | 930 | 1250 | 750 | 1023 | 300 |
| m^2^ per PE | 5 | 2.4 | 2.5 | 3.1 | 2.5 | 3.0 | 3.3 | 3.0 |
| **Filter Material** |  |  |  |  |  |  |  |  |
| Filter mineral | Fluv | Fluv | Fluv | Fluv | Cl-LS | Cl-LS | Ze-LS | Ze-LS |
| Geological origin | Rhine-Valley | Rhine-Valley | Rhine-Valley | Rhine-Valley | Eifel | Eifel | Eifel | Eifel |
| Grain size (mm) | 0.6-1.2 | 0.6-1.2 | 0.6-1.2 | 0.6-1.2 | 0-4 | 0-4 | 0-4 | 0-4 |
| BET surface area (m^2^ g^−1^) | 1.08 | 1.08 | 1.08 | 1.08 | 18.3 | 18.3 | 72.5 | 80.35 |
| **Laoding system** |  |  |  |  |  |  |  |  |
| Loading system | − | Pipe | − | − | Baffle plate | − | Baffle plate | Baffle plate |
| Baffle/ pipe density (m^−2^) | − | 2.25 | − | − | 12.5 | − | 25 | 25 |
| Loading rate (mm d^−1^) | − | 35 | − | − | 111 | − | 87 | 106 |

a: population equivalent; −: information unavailable

Fluv: fluviatile sand; Ze-LS : zeolite-dominated lava sand; Cl-LS: clinopyroxene-dominated lava sand

**Table S2.** Certified and measured concentrations of Cu, Fe, Mn Ni, Pb and Zn based on VBBo in certified reference materials LKSD-1 and LKSD-3.

|  | Cu (mg kg^−1^) | Fe (mg kg^−1^) | Mn (mg kg^−1^) | Ni (mg kg^−1^) | Pb (mg kg^−1^) | Zn (mg kg^−1^) |
| --- | --- | --- | --- | --- | --- | --- |
| *LKSD-1* |  |  |  |  |  |  |
| Certified | 4.4 | 1800 | 41 | 1.2 | 8.3 | 33.5 |
| Measured | 4.6 | 1950 | 39.5 | 1.3 | 7.7 | 33.1 |
| Recovery (%) | 96.3 | 108 | 103.8 | 94.9 | 107.5 | 101.2 |
| *LKSD-3* |  |  |  |  |  |  |
| Certified | 3.4 | 3600 | 130 | 4.6 | 2.1 | 15.1 |
| Measured | 3.4 | 3180 | 113 | 4.5 | 2.2 | 13.7 |
| Recovery (%) | 101.1 | 88.3 | 114.6 | 101.1 | 95.2 | 110.6 |

−: not determined

(c)

(d)

(a)

(b)

(e)

(f)

**Fig. S2.** Linear regression of (a) Fe, (b) Mn, (c) Zn, (d) Ni, (e) Pb and (f) Cu concentrations in fluviatile materials at different depths and operation time of construction wetlands, showing the methodic validation only for Cu and Pb.

**Table S3**, Metal budgets in fluviatile (Fluv) and zeolite- (Ze-LS) and clinopyroxene-dominant lava sand (Cl-LS) -based constructed wetlands to 60 cm deep

**Table S3-1.**

| **Fe** | Schnee-bergerhof | St. Alban | Würzweiler_1 | Würzweiler_2 | Tettingen-Butzdorf | Medelsheim | Riesweiler | Büschdorf |
| --- | --- | --- | --- | --- | --- | --- | --- | --- |
| Filter medium | Fluv | Fluv | Fluv | Fluv | Cl-LS | Cl-LS | Ze-LS | Ze-LS |
| Total background storage (g m^−2^) | 1160 ± 123 | 1160 ± 123 | 1160 ± 123 | 1160 ± 123 | 8960 ± 95 | 8960 ± 95 | 19600 ± 465 | 19600 ± 465 |
| Total Storage to date (g m^−2^) | 2070 ± 156 | 1830 ± 122 | 2360 ± 109 | 2120 ± 218 | 13700 ± 196 | 10200 ± 269 | 22400 ± 587 | 20500 ± 277 |
| Total net accumulation to date (g m^−2^) | 909 ± 199 | 674 ± 174 | 1200 ± 165 | 957 ± 251 | 4770 ± 284 | 1260 ± 284 | 2800 ± 750 | 858 ± 541 |
| Annual net accumulation (g m^−2^ yr^−1^) | 60.6 ± 13.3 | 74.9 ± 19.3 | 109 ± 15 | 137 ± 36 | 795 ± 36 | 209 ± 47 | 560 ± 150 | 123 ± 77 |
| Total adsorption capacity (pH 7.5) (g m^−2^) | − | − | − | − | − | − | − | − |
| Years for adsorption saturation expected (yr) | − | − | − | − | − | − | − | − |

−: not determined; The density of all filter materials are approximately 1.4 g cm^−3^.

**Table S3-2.**

| **Mn** | Schnee-bergerhof | St. Alban | Würzweiler_1 | Würzweiler_2 | Tettingen-Butzdorf | Medelsheim | Riesweiler | Büschdorf |
| --- | --- | --- | --- | --- | --- | --- | --- | --- |
| Filter medium | Fluv | Fluv | Fluv | Fluv | Cl-LS | Cl-LS | Ze-LS | Ze-LS |
| Total background storage (mg m^−2^) | 44200 ± 3192 | 44240 ± 3192 | 44240 ± 3192 | 44240 ± 3192 | 308000 ± 15800 | 308000 ± 15800 | 728000 ± 40300 | 728000 ± 40300 |
| Total Storage to date (mg m^−2^) | 47500 ± 889 | 18900 ± 295 | 92610 ± 1030 | 51500 ± 810 | 332000 ± 1620 | 235000 ± 1130 | 557000 ± 2270 | 585000 ± 1830 |
| Total net accumulation to date (mg m^−2^) | 3290 ± 3310 | -25300 ± 3210 | 48370 ± 3360 | 7224 ± 3290 | 23758 ± 15900 | -72500 ± 15900 | -17100 ± 40300 | -143000 ± 40300 |
| Annual net accumulation/ release (mg m^−2^ yr^−1^) | 219 ± 221 | -2810 ± 356 | 4400 ± 305 | 1030 ± 470 | 3960 ± 2660 | -12100 ± 2650 | -34200 ± 8070 | -20500 ± 5760 |
| Total adsorption capacity (pH 7.5) (mg m^−2^) | − | − | − | − | − | − | − | − |
| Years for adsorption saturation expected (yr) | − | − | − | − | − | − | − | − |

−: not determined; The density of all filter materials are approximately 1.4 g cm^−3^.

**Table S3-3.**

| **Zn** | Schnee-bergerhof | St. Alban | Würzweiler_1 | Würzweiler_2 | Tettingen-Butzdorf | Medelsheim | Riesweiler | Büschdorf |
| --- | --- | --- | --- | --- | --- | --- | --- | --- |
| Filter medium | Fluv | Fluv | Fluv | Fluv | Cl-LS | Cl-LS | Ze-LS | Ze-LS |
| Total background storage (mg m^−2^) | 6720 ± 235 | 6720 ± 235 | 6720 ± 235 | 6720 ± 235 | 30240 ± 4480 | 30240 ± 4480 | 36400 ± 5770 | 36400 ± 5770 |
| Total Storage to date (mg m^−2^) | 17800 ± 584 | 10800 ± 554 | 27000 ± 536 | 17700 ± 700 | 60600 ± 1090 | 45600 ± 454 | 47200 ± 534 | 70100 ± 908 |
| Total net accumulation to date (mg m^−2^) | 11100 ± 630 | 4080 ± 602 | 20300 ± 586 | 10900 ± 739 | 30400 ± 4610 | 15400 ± 4500 | 10800 ± 5790 | 33700 ± 5840 |
| Annual net accumulation (mg m^−2^ yr^−1^) | 739 ± 42 | 454 ± 67 | 186 ± 53 | 1560 ± 106 | 5060 ± 768 | 2560 ± 750 | 2170 ± 1160 | 4820 ± 834 |
| Total adsorption capacity (pH 7.5) (mg m^−2^) | 106400 | 106400 | 106400 | 106400 | 666400 | 666400 | 1240000 | 1240000 |
| Years for adsorption saturation expected (yr) | 144 | 234 | 57.6 | 68.0 | 132 | 260 | 574 | 258 |

−: not determined; The density of all filter materials are approximately 1.4 g cm^−3^.

**Table S3-4.**

| **Ni** | Schnee-bergerhof | St. Alban | Würzweiler_1 | Würzweiler_2 | Tettingen-Butzdorf | Medelsheim | Riesweiler | Büschdorf |
| --- | --- | --- | --- | --- | --- | --- | --- | --- |
| Filter medium | Fluv | Fluv | Fluv | Fluv | Cl-LS | Cl-LS | Ze-LS | Ze-LS |
| Total background storage (mg m^−2^) | 2800 ± 258 | 2800 ± 258 | 2800 ± 258 | 2800 ± 258 | 22960 ± 2910 | 22960 ± 2910 | 25200 ± 1400 | 25200 ± 1400 |
| Total storage to date (mg m^−2^) | 5120 ± 333 | 2970 ± 150 | 4300 ± 167 | 3270 ± 218 | 25800 ± 349 | 27500 ± 328 | 26800 ± 172 | 31900 ± 376 |
| Total net accumulation to date (mg m^−2^) | 2320 ± 421 | 166 ± 298 | 1500 ± 307 | 474 ± 338 | 2840 ± 2930 | 4530 ± 2930 | 1580 ± 1410 | 6730 ± 1450 |
| Annual net accumulation (mg m^−2^ yr^−1^) | 154 ± 28 | 18.4 ± 33.1 | 136 ± 28 | 67.7 ± 48.3 | 473 ± 488.8 | 755 ± 488 | 316 ± 282 | 961 ± 207 |
| Total adsorption capacity (pH 7.5) (mg m^−2^) | 33600 | 33600 | 33600 | 33600 | 252000 | 252000 | 448000 | 448000 |
| Years for adsorption saturation expected (yr) | 218 | 1820 | 247 | 496 | 532 | 334 | 1420 | 466 |

−: not determined; The density of all filter materials are approximately 1.4 g cm^−3^.

**Table S3-5.**

| **Cu** | Schnee-bergerhof | St. Alban | Würzweiler_1 | Würzweiler_2 | Tettingen-Butzdorf | Medelsheim | Riesweiler | Büschdorf |
| --- | --- | --- | --- | --- | --- | --- | --- | --- |
| Filter medium | Fluv | Fluv | Fluv | Fluv | Cl-LS | Cl-LS | Ze-LS | Ze-LS |
| Total background storage (mg m^−2^) | 1680 ± 140 | 1680 ± 140 | 1680 ± 140 | 1680 ± 140 | 25760 ± 728 | 25760 ± 728 | 71120 ± 2580 | 71120 ± 2580 |
| Total storage to date (mg m^−2^) | 6870 ± 395 | 3370 ± 275 | 5500 ± 270 | 3610 ± 316 | 30500 ± 451 | 27800 ± 436 | 74900 ± 547 | 72200 ± 453 |
| Total net accumulation to date (mg m^−2^) | 5190 ± 419 | 1690 ± 309 | 3820 ± 304 | 1930 ± 345 | 4720 ± 856 | 2030 ± 849 | 3750 ± 2630 | 1110 ± 2620 |
| Annual net accumulation (mg m^−2^ yr^−1^) | 346 ± 28 | 187 ± 34 | 348 ± 28 | 275 ± 49 | 787 ± 143 | 338 ± 141 | 751 ± 527 | 159 ± 373 |
| Total adsorption capacity (pH 7.5) (mg m^−2^) | 1736000 | 1736000 | 1736000 | 1736000 | 19936000 | 19936000 | 31900000 | 31900000 |
| Years for adsorption saturation expected (yr) | 5020 | 9270 | 4990 | 6310 | 25300 | 58900 | -2200000 | -469000 |

−: not determined; The density of all filter materials are approximately 1.4 g cm^−3^

**Table S3-6.**

| **Pb** | Schnee-bergerhof | St. Alban | Würzweiler_1 | Würzweiler_2 | Tettingen-Butzdorf | Medelsheim | Riesweiler | Büschdorf |
| --- | --- | --- | --- | --- | --- | --- | --- | --- |
| Filter medium | Fluv | Fluv | Fluv | Fluv | Cl-LS | Cl-LS | Ze-LS | Ze-LS |
| Total background storage (mg m^−2^) | 213 ± 39 | 213 ± 39 | 213 ± 39 | 213 ± 39 | 1860 ± 56 | 1860 ± 56 | 2540 ± 129 | 2540 ± 129 |
| Total storage to date (mg m^−2^) | 922 ± 113 | 443 ± 28 | 602 ± 38 | 450 ± 57 | 2350 ± 115 | 2680 ± 31 | 2920 ± 54 | 2920 ± 48 |
| Total net accumulation to date (mg m^−2^) | 709 ± 132 | 231 ± 48 | 389 ± 54 | 238 ± 68 | 489 ± 128 | 814 ± 63 | 386 ± 139 | 386 ± 137 |
| Annual net accumulation (mg m^−2^ yr^−1^) | 47.3 ± 8.0 | 25.6 ± 5.3 | 35.4 ± 4.9 | 34.0 ± 9.8 | 81.6 ± 21.38 | 136 ± 10.7 | 77.1 ± 27.9 | 55.1 ± 19.6 |
| Total adsorption capacity (pH 7.5) (mg m^−2^) | − | − | − | − | − | − | − | − |
| Years for adsorption saturation expected (yr) | − | − | − | − | − | − | − | − |

−: not determined; The density of all filter materials are approximately 1.4 g cm^−3^

**Table S4.** Calculated annual accumulation or release rates of Fe, Mn, Zn, Ni, Cu and Pb at different depths of constructed wetland profiles based on the concentration differences of metals in original filter materials or the deepest fluviatile layers, filter materials sampled from constructed wetlands and the operation time.

**Table S4-1.**

| **Fe (g kg^−1^ yr^−1^)** |  |  |  |  |  |  |  |  |  |  |
| --- | --- | --- | --- | --- | --- | --- | --- | --- | --- | --- |
| **Site** | **Filter** | **0-10 cm** | | | **10-20 cm** | | | **30-40 cm** | | |
| Schneebergerhof | Fluv | 0.15 | ± | 0.05 | 0.13 | ± | 0.07 | 0.06 | ± | 0.02 |
| St. Alban | Fluv | 0.32 | ± | 0.09 | 0.08 | ± | 0.02 | 0.06 | ± | 0.03 |
| Würzweiler_1 | Fluv | 0.38 | ± | 0.05 | 0.15 | ± | 0.03 | 0.12 | ± | 0.02 |
| Würzweiler_2 | Fluv | 0.79 | ± | 0.35 | 0.08 | ± | 0.03 | 0.05 | ± | 0.03 |
| Tettingen-Butzdorf | Cl-LS | 1.38 | ± | 0.12 | 1.48 | ± | 0.13 | 1.38 | ± | 0.28 |
| Medelsheim | Cl-LS | 0.41 | ± | 0.38 | 0.46 | ± | 0.34 | 0.27 | ± | 0.33 |
| Riesweiler | Ze-LS | -0.05 | ± | 3.50 | 1.38 | ± | 0.28 | 1.32 | ± | 0.28 |
| Büschdorf | Ze-LS | 0.54 | ± | 0.25 | 0.40 | ± | 0.30 | -0.17 | ± | 0.45 |

Fluv: fluviatile sand; Cl-LS: clinopyroxene-dominated lava sand; Ze-LS : zeolite-dominated lava sand

**Table S4-2.**

| **Mn (mg kg^−1^ yr^−1^)** |  |  |  |  |  |  |  |  |  |  |
| --- | --- | --- | --- | --- | --- | --- | --- | --- | --- | --- |
| **Site** | **Filter** | **0-10 cm** | | | **10-20 cm** | | | **30-40 cm** | | |
| Schneebergerhof | Fluv | 2.47 | ± | 2.04 | -1.13 | ± | 1.84 | 0.53 | ± | 0.38 |
| St. Alban | Fluv | -3.94 | ± | 0.77 | -5.89 | ± | 0.67 | -4.89 | ± | 0.63 |
| Würzweiler_1 | Fluv | 22.0 | ± | 4.57 | 3.64 | ± | 2.07 | 2.64 | ± | 0.52 |
| Würzweiler_2 | Fluv | 16.1 | ± | 4.78 | -3.14 | ± | 1.13 | -2.69 | ± | 0.81 |
| Tettingen-Butzdorf | Cl-LS | 28.8 | ± | 21.2 | 1.83 | ± | 6.41 | -2.17 | ± | 8.44 |
| Medelsheim | Cl-LS | -21.4 | ± | 7.76 | -23.7 | ± | 10.0 | -19.7 | ± | 4.72 |
| Riesweiler | Ze-LS | -121 | ± | 27.6 | -61.3 | ± | 49.3 | -21.1 | ± | 14.4 |
| Büschdorf | Ze-LS | -31.9 | ± | 18.7 | -33.7 | ± | 21.5 | -42.4 | ± | 10.3 |

Fluv: fluviatile sand; Cl-LS: clinopyroxene-dominated lava sand; Ze-LS : zeolite-dominated lava sand

**Table S4-3.**

| **Zn (mg kg^−1^ yr^−1^)** |  |  |  |  |  |  |  |  |  |  |
| --- | --- | --- | --- | --- | --- | --- | --- | --- | --- | --- |
| **Site** | **Filter** | **0-10 cm** | | | **10-20 cm** | | | **30-40 cm** | | |
| Schneebergerhof | Fluv | 3.63 | ± | 1.13 | 0.75 | ± | 0.24 | 0.35 | ± | 0.11 |
| St. Alban | Fluv | 3.13 | ± | 1.67 | 0.44 | ± | 0.49 | -0.36 | ± | 0.11 |
| Würzweiler_1 | Fluv | 9.19 | ± | 1.22 | 2.09 | ± | 0.51 | 0.57 | ± | 0.19 |
| Würzweiler_2 | Fluv | 9.43 | ± | 3.57 | 1.02 | ± | 0.22 | 0.14 | ± | 0.04 |
| Tettingen-Butzdorf | Cl-LS | 22.6 | ± | 7.64 | 8.99 | ± | 6.12 | 0.02 | ± | 3.23 |
| Medelsheim | Cl-LS | 10.4 | ± | 2.01 | 5.70 | ± | 1.54 | -0.42 | ± | 1.41 |
| Riesweiler | Ze-LS | 8.63 | ± | 3.01 | 3.43 | ± | 2.32 | 1.14 | ± | 2.06 |
| Büschdorf | Ze-LS | 19.4 | ± | 5.67 | 6.55 | ± | 2.83 | 3.43 | ± | 1.55 |

Fluv: fluviatile sand; Cl-LS: clinopyroxene-dominated lava sand; Ze-LS : zeolite-dominated lava sand

**Table S4-4.**

| **Ni (mg kg^−1^ yr^−1^)** |  |  |  |  |  |  |  |  |  |  |
| --- | --- | --- | --- | --- | --- | --- | --- | --- | --- | --- |
| **Site** | **Filter** | **0-10 cm** | | | **10-20 cm** | | | **30-40 cm** | | |
| Schneebergerhof | Fluv | 0.56 | ± | 0.15 | 0.37 | ± | 0.35 | 0.07 | ± | 0.04 |
| St. Alban | Fluv | 0.36 | ± | 0.14 | 0.01 | ± | 0.01 | -0.02 | ± | 0.02 |
| Würzweiler_1 | Fluv | 0.68 | ± | 0.13 | 0.22 | ± | 0.03 | 0.10 | ± | 0.04 |
| Würzweiler_2 | Fluv | 0.84 | ± | 0.35 | 0.00 | ± | 0.01 | -0.05 | ± | 0.02 |
| Tettingen-Butzdorf | Cl-LS | 0.91 | ± | 0.99 | 0.78 | ± | 0.96 | 0.86 | ± | 1.20 |
| Medelsheim | Cl-LS | 0.78 | ± | 0.88 | 1.58 | ± | 1.13 | 1.49 | ± | 1.02 |
| Riesweiler | Ze-LS | 1.21 | ± | 0.53 | 0.48 | ± | 0.56 | 0.22 | ± | 0.05 |
| Büschdorf | Ze-LS | 2.08 | ± | 0.60 | 1.62 | ± | 0.67 | 1.57 | ± | 0.79 |

Fluv: fluviatile sand; Clinopyroxene-LS: clinopyroxene-dominated lava sand; Ze-LS : zeolite-dominated lava sand

**Table S4-5.**

| **Cu (mg kg^−1^ yr^−1^)** |  |  |  |  |  |  |  |  |  |  |
| --- | --- | --- | --- | --- | --- | --- | --- | --- | --- | --- |
| **Site** | **Filter** | **0-10 cm** | | | **10-20 cm** | | | **30-40 cm** | | |
| Schneebergerhof | Fluv | 1.64 | ± | 0.42 | 0.49 | ± | 0.32 | 0.07 | ± | 0.04 |
| St. Alban | Fluv | 1.06 | ± | 0.42 | 0.24 | ± | 0.07 | -0.05 | ± | 0.03 |
| Würzweiler_1 | Fluv | 1.77 | ± | 0.32 | 0.42 | ± | 0.09 | 0.06 | ± | 0.04 |
| Würzweiler_2 | Fluv | 1.97 | ± | 0.72 | 0.16 | ± | 0.07 | -0.16 | ± | 0.05 |
| Tettingen-Butzdorf | Cl-LS | 4.30 | ± | 1.39 | 1.10 | ± | 0.85 | -0.22 | ± | 0.70 |
| Medelsheim | Cl-LS | 1.33 | ± | 0.81 | 0.56 | ± | 1.23 | 0.16 | ± | 0.77 |
| Riesweiler | Ze-LS | 1.61 | ± | 1.84 | 1.11 | ± | 1.86 | 1.39 | ± | 2.24 |
| Büschdorf | Ze-LS | 0.60 | ± | 0.78 | 0.24 | ± | 1.09 | 0.12 | ± | 1.32 |

Fluv: fluviatile sand; Cl-LS: clinopyroxene-dominated lava sand; Ze-LS : zeolite-dominated lava sand

**Table S4-6.**

| **Pb (mg kg^−1^ yr^−1^)** |  |  |  |  |  |  |  |  |  |  |
| --- | --- | --- | --- | --- | --- | --- | --- | --- | --- | --- |
| **Site** | **Filter** | **0-10 cm** | | | **10-20 cm** | | | **30-40 cm** | | |
| Schneebergerhof | Fluv | 0.22 | ± | 0.05 | 0.06 | ± | 0.02 | 0.02 | ± | 0.01 |
| St. Alban | Fluv | 0.08 | ± | 0.02 | 0.03 | ± | 0.01 | 0.03 | ± | 0.01 |
| Würzweiler_1 | Fluv | 0.14 | ± | 0.02 | 0.05 | ± | 0.01 | 0.02 | ± | 0.01 |
| Würzweiler_2 | Fluv | 0.17 | ± | 0.06 | 0.03 | ± | 0.01 | 0.02 | ± | 0.01 |
| Tettingen-Butzdorf | Cl-LS | 0.40 | ± | 0.08 | 0.10 | ± | 0.08 | 0.02 | ± | 0.08 |
| Medelsheim | Cl-LS | 0.46 | ± | 0.03 | 0.26 | ± | 0.02 | 0.08 | ± | 0.03 |
| Riesweiler | Ze-LS | 0.34 | ± | 0.06 | 0.09 | ± | 0.05 | 0.04 | ± | 0.08 |
| Büschdorf | Ze-LS | 0.27 | ± | 0.05 | 0.05 | ± | 0.04 | 0.03 | ± | 0.04 |

Fluv: fluviatile sand; Cl-LS: clinopyroxene-dominated lava sand; Ze-LS : zeolite-dominated lava sand
